# Dietary and microbiome factors determine longevity in *Caenorhabditis elegans*

**DOI:** 10.18632/aging.101008

**Published:** 2016-07-31

**Authors:** Adolfo Sánchez-Blanco, Alberto Rodríguez-Matellán, Ana González-Paramás, Susana González-Manzano, Stuart K. Kim, Faustino Mollinedo

**Affiliations:** ^1^ Instituto de Biología Molecular y Celular del Cáncer, Centro de Investigación del Cáncer, CSIC-Universidad de Salamanca, Campus Miguel de Unamuno, E-37007 Salamanca, Spain; ^2^ Grupo de Investigación en Polifenoles, Facultad de Farmacia, Unidad de Nutrición y Bromatología, Universidad de Salamanca, E-37007 Salamanca, Spain; ^3^ Departments of Developmental Biology and Genetics, Stanford University, Stanford, CA 94305, USA; ^4^ Current address: Department of Biology, University of Hartford, West Hartford, CT 06117, USA; ^5^ Current address: Laboratory of Cell Death and Cancer Therapy, Department of Cellular and Molecular Medicine, Centro de Investigaciones Biológicas, Consejo Superior de Investigaciones Científicas (CSIC), E-28040 Madrid, Spain; ^6^ Current address: Centro de Biología Molecular Severo Ochoa (CSIC-UAM), E‐28049 Madrid, Spain

**Keywords:** C. elegans, longevity, microbiome, oxidants, coenzyme Q, redox homeostasis

## Abstract

Diet composition affects organismal health. Nutrient uptake depends on the microbiome. *Caenorhabditis elegans* fed a *Bacillus subtilis* diet live longer than those fed the standard *Escherichia coli* diet. Here we report that this longevity difference is primarily caused by dietary coQ, an antioxidant synthesized by *E. coli* but not by *B. subtilis*. CoQ-supplemented *E. coli* fed worms have a lower oxidation state yet live shorter than coQ-less *B. subtilis* fed worms. We showed that mutations affecting longevity for *E. coli* fed worms do not always lead to similar effects when worms are fed *B. subtilis*. We propose that coQ supplementation by the *E. coli* diet alters the worm cellular REDOX homeostasis, thus decreasing longevity. Our results highlight the importance of microbiome factors in longevity, argue that antioxidant supplementation can be detrimental, and suggest that the *C. elegans* standard *E. coli* diet can alter the effect of signaling pathways on longevity.

## INTRODUCTION

Diet quality is correlated with human health and life span. However, the complexity of variables involved in dietary patterns makes it extremely difficult to evaluate the importance that specific diet inputs exert on health and life expectancy [1]. The simplicity of model organisms like *C. elegans* can be very helpful to understand how dietary factors may impact complex traits such as development, physiology, metabolism or aging.

*C. elegans* is a bacteriovore and in the laboratory its standard diet is *Escherichia coli* OP50 [2]. Replacing *E*. *coli* with other bacterial monocultures allows for a straightforward way to investigate the impact that species-specific nutrients can have on different aspects of biology. An example of the usefulness of *C. elegans* in understanding the complex interactions between diet and physiology comes from feeding worms *Comamonas* DA1877 instead of *E. coli*. *Comamonas* diet changes *C. elegans* gene expression patterns and alters the life history traits of the worm provoking accelerated development, reduction in progeny, and life span shortening [3,4]. *Comamonas* provide worms with vitamin B12, which is responsible for many of the *C. elegans* expression changes as well as for the accelerated development and fertility reduction [5]. The mechanism of vitamin B12 activity in *C. elegans* represents a valuable model to understand and characterize vitamin B12-dependent processes as well as to provide potential treatments to vitamin B12-related deficiencies in humans [5].

A great advantage of using *C. elegans* to study how dietary compounds affect complex biological traits resides in the ease of genetic manipulation of *C. elegans* and its bacterial diet. For instance, Maier et al. reported that different *E. coli* strains affected worm life span differently and that the longevity effects were modulated by different subsets of sensory neurons via *nmur-1*, a homolog of mammalian neuromedin U receptors. Using *E. coli* mutant strains, they determined that the *nmur-1* life span effect was dependent on the type of *E. coli* lipopolysaccharide structure [6]. By combining *C. elegans* and *E. coli* genetics, another study showed that excessive dietary folate from *E. coli* negatively influenced *C. elegans* longevity, thus identifying microbial folate synthesis as a potential target to slow animal aging pharmacologically [7]. Previously, Larsen and Clarke observed that worms considerably increased their life span when fed mutated *E. coli* strains that were unable to synthesize coenzyme Q (coQ) [8]. It has been proposed that the *C. elegans* longevity increase when worms are fed coQ-defective *E. coli* is due to alterations in *E. coli* respiration induced by the lack of coQ, which in turn delay the accumulation of *E. coli* in the worm intestine [9,10].

*C. elegans* bacterivory provides an advantageous model to understand the complex dynamics that the worm establishes with its bacterial prey through host–microbiota interactions. The intersection between *C. elegans* diet and microbiota is an extremely valuable asset to understand the importance that the microbiome can have to different aspects of the biology of its host [11–13]. For example, Cabreiro et al. revealed that the longevity increase of *C. elegans* life span upon treatment with metformin, a drug commonly used to treat type 2 diabetes, is due to alterations to *E. coli* folate and methionine metabolism. These findings highlight the interaction that the microbiome can have on pharmacological therapies [14].

While used as the standard laboratory *C. elegans* diet, *E. coli* is not a type of food that worms would normally encounter in the wild [15,16]. *C. elegans* genetic and physiological behavior when confronted with other diets that are more likely to be part of its natural microbiome has been the focus of interest of a number of studies. Garsin et al. reported that worms lived longer when fed the gram positive bacterium *Bacillus subtilis* instead of *E. coli* [17]. Similarly, worms fed *Bacillus megaterium* lived longer than *E. coli* fed worms [15]. *Bacillus* species are commonly found in the natural *C. elegans* soil environment and as part of the *C. elegans* microbiome, and have been shown to procure *C. elegans* with enhanced resistance against infection with the pathogen *Pseudomonas aeruginosa*, suggesting a stimulation of the innate immunity by the *C. elegans* microbiota [16].

*C. elegans* lacks nitric oxide synthases to produce their own nitric oxide, a signaling molecule involved in many biological processes. Unlike *E. coli*, *B. subtilis* and other bacteria of the *C. elegans* soil habitat are able to generate nitric oxide. It was shown that nitric oxide derived from *B. subtilis* contributes to *C. elegans* longevity and stress resistance. This work exemplifies the coevolution of *C. elegans* with its microbiome and raises the possibility of the beneficial properties that nitric oxide from intestinal microbiota may produce to humans [18].

Feeding *B. subtilis* to *C. elegans* instead of *E. coli* not only alters their longevity but these diets appear to induce different causes of death to the worms [19]. Pathophysiological studies revealed that the intestine of *E. coli* fed worms suffered a major decline during aging [20]. Interestingly, intestinal pathogenicity has been suggested as a major cause of death for *E. coli* fed worms [19]. We wanted to explore the dietary and microbiome factors that contribute to the life span difference when worms are fed *B. subtilis* instead of *E. coli*. We showed that lack of the antioxidant coQ in *B. subtilis* is a major factor that explains the life span difference between *E. coli* and *B. subtilis* fed worms. CoQ-supplemented *E. coli* fed worms are able to better counteract the effect of the oxidant paraquat during development than *B. subtilis* fed worms as they have lower cellular ROS and lipid peroxidation levels than *B. subtilis* fed worms. We propose that the lifelong antioxidant effect of coQ supplementation by the *E. coli* diet provokes alterations in the worm cellular REDOX homeostasis, which in turn lead to *C. elegans* life span shortening. We also showed that genetic interventions that affect the life span of *E. coli* fed worms do not always lead to similar effects when worms are fed *B. subtilis*. Our results highlight the importance of the microbiome in longevity and argue that antioxidant supplementation can be detrimental. Our work also suggests that using *E. coli* as standard *C. elegans* diet may alter the effect that a given signaling pathway can have on longevity.

## RESULTS

### *B. subtilis* fed worms live longer and die because of different reasons than *E. coli* fed worms

**Figure 1:**
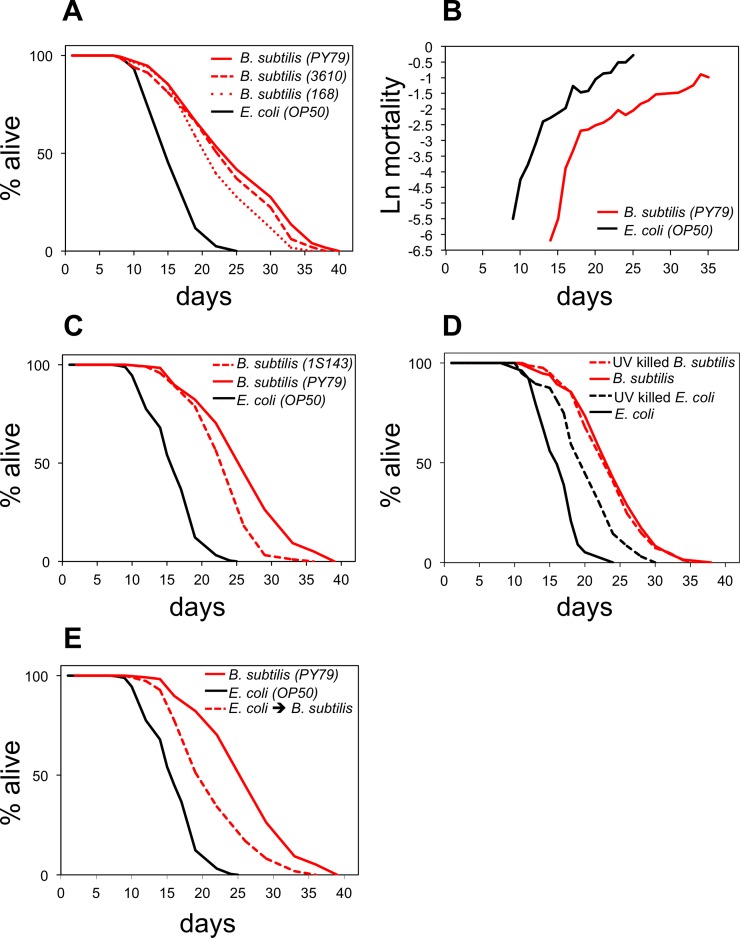
*B. subtilis* fed worms live longer and die because of different reasons than *E. coli* fed worms (**A**) Life span curves for adult worms maintained on *E. coli* (OP50) or on different wild type strains of *B. subtilis*: PY79, 3610, and 168. (**B**) Age-related mortality for worms fed *B. subtilis* or *E. coli* (see also Figure S2 for life span and death frequency over time data). (**C**) Life span curves for adult worms maintained on *E. coli*, *B. subtilis*, or the sporeless *B. subtilis* mutant (1S143). (**D**) Life span curves for adult worms maintained on live *E. coli*, UV-killed *E. coli*, live *B. subtilis*, or UV-killed *B. subtilis*. (**E**) Life span curves for adult worms maintained on *E. coli*; on *B. subtilis*; or on *E. coli* as late L4 stage and then switched to *B. subtilis*. (**A**-**E**) y-axis indicates percentage of worms that are alive. x-axis indicates day of adulthood. See also Table S1.

Feeding the gram positive *B. subtilis* to *C. elegans* instead of the gram negative standard laboratory diet *E. coli* considerably increases worm longevity [14,17,19,21]. To understand the nature of this life span increase we first examined whether it is *B. subtilis* strain-specific. We fed worms three different WT strains of *B. subtilis* (PY79, 3610, and 168) and compared their longevity to worms fed the standard *E. coli* OP50. Worms fed any of the *B. subtilis* strains lived longer (43%-58%) than *E. coli* fed worms (Figure 1A), thus feeding *B. subtilis* to worms extends their life span independently of the *B. subtilis* strain used.

To investigate the overall health of *B. subtilis* fed worms, we compared *C. elegans* gut autofluorescence accumulation and muscle decline during aging for *B. subtilis* and *E. coli* fed worms. Age-related fluorescent breakdown products accumulate in the gut during aging leading to increasing levels of autofluorescence with age [19]. *B. subtilis* fed worms displayed less gut autofluorescence late in life than *E. coli* fed worms (Figure S1A). UNC-54 is the major myosin heavy chain expressed in *C. elegans* and is required for *C. elegans* locomotion [22]. We used a *unc-54::mCherry* reporter *C. elegans* strain [19] to measure muscle decline and observed a lower decline of *unc-54* expression in *B. subtilis* fed worms than in *E. coli* fed worms during aging (Figure S1B). These data indicate that the overall health of *B. subtilis* fed worms declines at a slower rate during aging than that of *E. coli* fed worms.

Previous work indicated that factors associated with the *E. coli* and the *B. subtilis* diets make worms die because of different reasons [19]. To further investigate the underlying mechanism, we performed demographic longevity studies in which we subjected 500 individuals to each diet and examined their mortality rates and death frequency over time (Figure 1B; Figure S2). We observed a lowering of the initial mortality rate but not a major impact on the rate of aging when worms were fed *B. subtilis* as compared to *E. coli*. This suggests that feeding worms *E. coli* would add additional risk factors to the worms at all ages or that feeding worms *B. subtilis* would eliminate risk factors at all ages.

*B. subtilis* are able to sporulate while *E. coli* are not. Spores are not digestible and upon germination might produce factors that affect worm life span. We tested this possibility by using a *B. subtilis* strain that is unable to sporulate. Worms fed this spore-less *B. subtilis* diet lived 46% longer than *E. coli* fed worms (Figure 1C). Although *B. subtilis* spore presence increased worm longevity by an additional 10% (Figure 1C), the data show that the life span difference between *E. coli* and *B. subtilis* fed worms is not solely due to the presence of *B. subtilis* spores.

Since life span studies are usually performed with live bacteria, one possibility is that worms that feed on *E. coli* are more prone to infection than worms that feed on *B. subtilis*. Bacteria that are not properly processed before being digested can enter the worm gut and proliferate [23]. *E. coli* proliferation has been shown to be harmful to *C. elegans* [24]. Therefore, excessive bacterial gut proliferation could be the cause of the life span shortening when worms are fed *E. coli* instead of *B. subtilis*. We compared the longevity of worms fed either live *E. coli* or live *B. subtilis versus* non-infectious UV killed *E. coli* or UV killed *B. subtilis*. Worms fed live or UV killed *B. subtilis* had very similar life spans (Figure 1D). However, as previously reported [19,25], worms fed UV killed *E. coli* lived approximately 20% longer than worms fed live *E. coli* (Figure 1D), indicating that *E. coli* infection is a contributing factor for the shortening of the life span when worms are fed *E. coli*. However, *E. coli* infection does not explain the overall life span increase observed when worms are fed *B. subtilis* instead of *E. coli* as worms that feed on *B. subtilis* (live or UV killed) live longer than UV killed *E. coli* fed worms.

To examine whether *E. coli* fed worms live shorter than *B. subtilis* fed worms because of *E. coli*-specific pathogenic factors, we fed worms *E. coli* only during development (L1 to late L4) and then changed their diet to *B. subtilis* for the remainder of their lives. These worms lived 30% shorter than worms that were exclusively fed on *B. subtilis* (Figure 1E) suggesting that *E. coli* fed worms are exposed to negative effects by *E. coli*, which shorten *C. elegans* longevity.

### *B. subtilis* do not induce dietary restriction to *C. elegans*

Dietary restriction has been shown to extend *C. elegans* life span [26,27]. If the *B. subtilis* diet induced a state of dietary restriction to the worms, then it would explain the longevity difference with respect to *E. coli* fed worms. We determined that the caloric content of *B. subtilis* and *E. coli* is very similar (Table S2). We also showed that these two types of bacteria have very similar water content and that the major calorie contribution is conferred by protein and carbohydrate.

**Figure 2:**
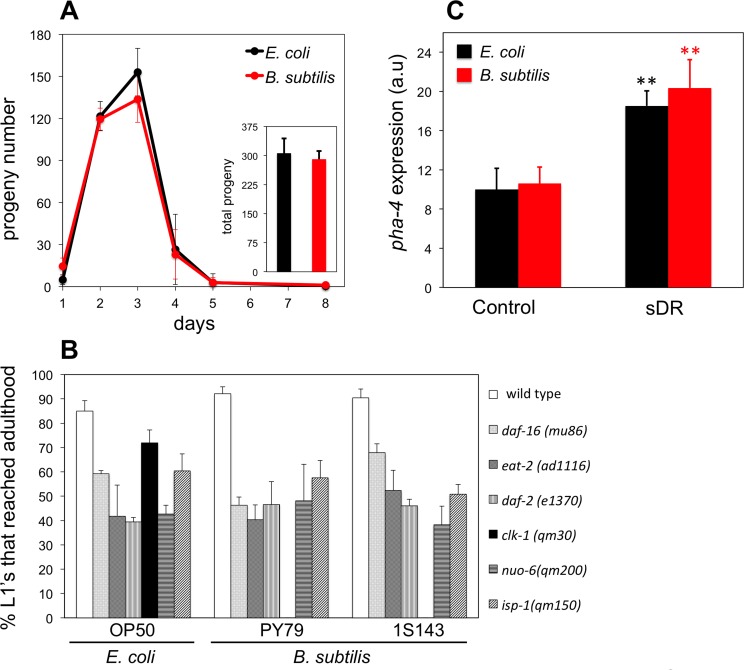
*B. subtilis* fed worms are not dietary restricted (**A**) Daily average number of progeny ± S.E.M per worm and diet (n = 5 for each group). Inset bars represent the total average progeny ± S.E.M per worm on *E. coli* and *B. subtilis*. y-axis shows progeny number. x-axis shows age of adult worms. No statistical differences for each diet and group were observed (p > 0.05, Student's t test). (**B**) Percentage of L1 larvae that became adults when fed *E. coli* or *B. subtilis*, wild type (PY79) and spore-less mutant (1S143), after 2.7 days (wild type worms), 2.7 days (*daf-16* worms), 2.9 days (*eat-2* worms), 3.6 days (*daf-2* worms), 3.2 days (*clk-1* worms), 5.0 days (*nuo-6* worms), 5.0 days (*isp-1* worms). y-axis shows percentage of L1 larvae that reached adulthood. x-axis shows *E. coli* or *B. subtilis* fed worms. n = 44-160 worms per group. Bars indicate the mean value ± S.E.M. No statistical differences for each diet and group were observed (p > 0.05, Student's t test) except for *clk-1* worms. (**C**) *pha-4::mCherry* fluorescent marker expression for 2 day old adults exposed to plenty of food (Control) or dietary restricted (sDR). y-axis shows levels of fluorescent expression in arbitrary units. x-axis shows *E. coli* or *B. subtilis* fed worms. Bars indicate mean fluorescent marker expression ± S.E.M. n = 10-20 for each group (**p < 0.01, Student's t test).

It has been shown that *C. elegans* do not display noticeable feeding behavior modifications when fed *B. subtilis* and that these bacteria are efficiently ingested and digested by *C. elegans* (Laaberki and Dworkin, 2008b). Independently, we also tested whether *B. subtilis* cells might not be well digested by *C. elegans*, thus not allowing worms to get the same amount of nourishment as worms that feed on *E. coli*. First, we examined markers of worm metabolic activity. Egg production and developmental growth are high-energy demanding metabolic processes and thus good indicators of the metabolic status of the worm. Interventions that compromise *C. elegans* metabolism such as dietary restriction lead to reduced brood size and/or delayed development [25,28–30]. We monitored the daily and the overall brood size of *B. subtilis* and *E. coli* fed worms but did not observe any differences between the two feeding conditions (Figure 2A). We compared the percentage of WT L1 larvae that developed to adults after 2.7 days of feeding *E. coli* or two different strains of *B. subtilis* and, similar to previously published results [17,29] we did not see any differences (Figure 2B). We also compared the percentage of L1 larvae that developed to adults when worms were fed *E. coli* or *B. subtilis* for a number of *C. elegans* mutants known to develop slower. These mutant strains included the *daf-2* insulin-like growth factor 1 receptor; the *daf-16* forkhead box O (FOXO) homologue; the *isp-1* and *nuo-6* mitochondrial electron transport chain subunit components; the *eat-2* nicotinic acetylcholine receptor subunit*,* which mimics dietary restriction; and the coQ-deficient *clk-1*. With the exception of *clk-1* worms, we did not see differences in the percentage of L1 larvae that developed to adults when these mutant worms were fed either diet (Figure 2B).

We also used a transgenic *pha-4::mCherry* trans-criptional reporter strain [19], which can indicate the nutritional state of worms as it displays elevated fluorescence expression upon dietary restriction conditions (Figure S3). PHA-4 is a Foxa transcription factor that has been shown to play an important role in the *C. elegans* starvation response [31] as well as to mediate diet-restriction-induced longevity in *C. elegans* [32]. We found that *ad libitum E. coli* and *B. subtilis* fed worms displayed the same *pha-4::mCherry* expression levels (Figure 2C). We also found that *E. coli* or *B. subtilis* fed worms that were subjected to dietary restriction increased *pha-4::mCherry* expression. Importantly, the increase in *pha-4::mCherry* expression was similar regardless of the diet type (Figure 2C).

Three independent lines of evidence including the similar caloric content for *B. subtilis* and *E. coli*; the equal progeny number and developmental time to reach adulthood for worms fed *B. subtilis* or *E. coli* ; and the *pha-4::mCherry* nutritional biomarker response for worms fed either diet indicate that *B. subtilis* fed worms are not under the effects of dietary restriction and thus their longevity increase compared to *E. coli* fed worms must be due to some other reason.

### Worms fed *B. subtilis* instead of *E. coli* live longer mainly because this food source does not contain coenzyme Q

CoQ-deficient *clk-1* larvae were unable to develop when fed *B. subtilis* (Figure 2B). We confirmed this result using another *clk-1* allele (Figure S4). *B. subtilis* fed *clk-1* L1 larvae developed into L2 stage and arrested. After approximately one week, most L2 larvae continued their development to adulthood but worms were sterile. We maintained *clk-1* larvae on *B. subtilis* for up to 30 days but these still failed to develop into fertile adults. However, if arrested larvae were transferred to *E. coli* plates, development resumed and larvae became fertile adults. Conversely, fertile *E. coli* fed *clk-1* young adults became sterile 2 days upon transfer to *B. subtilis* plates.

CoQ is a crucial mitochondrial electron transport chain (ETC) carrier [33]. *C. elegans* synthesize their own coQ (Q_9_) and the demethoxyubiquinone hydroxylase CLK-1 is a necessary enzyme in this process. Thus, *clk-1* mutants are Q_9_ deficient [34]. If worms completely lack coQ, then they become sterile and larvae are not able to complete development [35,36]. Although *clk-1* mutant worms cannot synthesize Q_9_, they uptake coQ (Q_8_) from the *E. coli* diet, which explains why under standard laboratory conditions *clk-1* worms can develop and function [36,37]. Unlike *E. coli*, *B. subtilis* do not synthesize coQ [38]. Therefore, feeding coQ-less *B. subtilis* to coQ-deficient *clk-1* worms leads to developmental arrest and adult sterility. These phenotypes are comparable to those observed when *clk-1* worms are fed coQ-deficient *E. coli* [35–37,39]. Worms fed coQ-deficient *E. coli* mutant strains live up to 60% longer than coQ-producing *E. coli* fed worms [8]. CoQ-less *B. subtilis* fed worms also live much longer than coQ-producing *E. coli* fed worms [17] (Figure 1). Therefore, we wanted to investigate whether the extended longevity of *B. subtilis* versus *E. coli* fed worms was due to their lack of dietary coQ.

**Figure 3:**
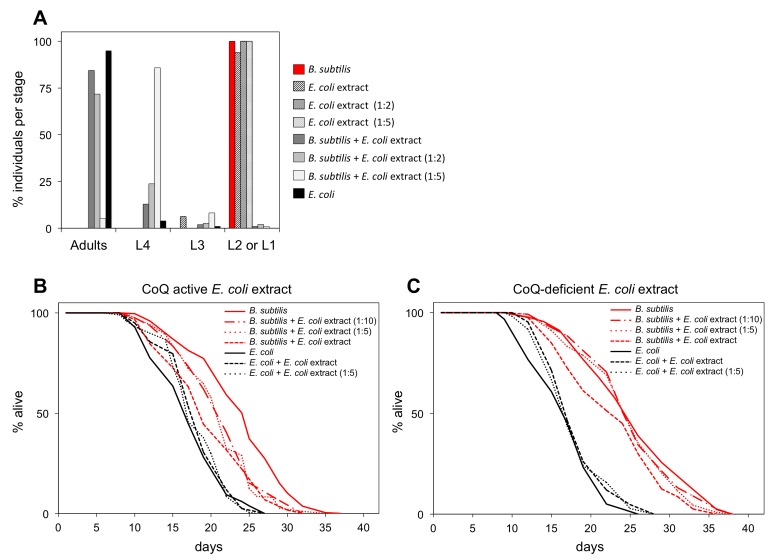
*B. subtilis* fed worms supplemented with coQ-active *E. coli* extract have shortened longevity (**A**) Development of synchronized *clk-1(qm30)* L1 larvae after 4.7 days feeding on *E. coli*, *E. coli* extract at different concentrations, or *B. subtilis* with or without supplementation of *E. coli* extract at different concentrations. y-axis shows percentage of individuals that reached each developmental stage. x-axis shows developmental stages. n= 51-160 worms per group (**B**) Life span curves for adult worms maintained on *E. coli* or *B. subtilis* with or without supplementation of coQ-active *E. coli* (OP50) extract at different concentrations. (**C**) Life span curves for adult worms maintained on *E. coli* or *B. subtilis* with or without supplementation of coQ-deficient *E. coli* (GD1) extract at different concentrations. (**B** and **C**) y-axis indicates percentage of worms that are alive. x-axis indicates day of adulthood. See also Table S4.

We compared the life span of *B. subtilis* fed worms with the life span of *B. subtilis* fed worms supplemented with coQ. CoQ is highly hydrophobic, thus supplementation of synthetic coQ to the aqueous *C. elegans* media makes it difficult for worms to incorporate this compound [10]. To ensure an effective level of coQ supplementation as well as to supplement with the same coQ species worms receive when they feed on the standard *E. coli* diet, we developed a coQ supplementation method using non-nutritious *E. coli* extracts that retained coQ activity (see Experimental Procedures). CoQ-deficient *clk-1* L1 larvae reared on the coQ-less *B. subtilis* diet were able to develop when the *E. coli* extract was present, demonstrating that the *E. coli* extract retained coQ activity. The rate of *clk-1* L1 to adult development was proportional to the concentration of *E. coli* extract used (Figure 3A) and the adult sterility when *clk-1* worms were fed *B. subtilis* was reversed when the *E. coli* extract was added (Table S3). Importantly, the *E. coli* extract was not nutritious as *clk-1* L1 larvae that are solely fed with it arrested at L1 stage (Figure 3A).

We supplemented *B. subtilis* fed worms with non-nutritious coQ-active *E. coli* extract. These worms had a shorter life span than *B. subtilis* fed worms. This life span shortening effect is dose-dependent upon the concentration of coQ-active *E. coli* extract (Figure 3B). Importantly, supplementation of *B. subtilis* fed worms with coQ-deficient *E. coli* extract led only to a minor life span decrease compared to worms fed exclusively on *B. subtilis* (Figure 3C). *E. coli* extracts likely contain other components besides coQ that are detrimental to the worm such as lipopolysaccharides (LPS) (Maier et al., 2010), folic acid (Virk et al., 2012), etc. Therefore, the minor life span decrease observed when *B. subtilis* fed worms are supplemented with coQ-deficient *E. coli* extract is likely due to the presence of these other *E. coli* components. The fact that coQ-active *E. coli* extracts significantly decreased the life span extension of *B. subtilis* fed worms whereas coQ-deficient *E. coli* extracts only led to a slight life span decrease indicates that the contribution of other factors besides coQ from the *E. coli* extract to the life span shortening of *B. subtilis* fed worms is smaller, thus suggesting that *B. subtilis* fed worms live longer mainly because this diet does not provide supplemental coQ to the worms.

*E. coli* fed worms to which coQ-active *E. coli* extracts were added lived the same as *E. coli* fed worms (Figure 3B). Moreover, *E. coli* fed worms to which coQ-less extracts from either mutant *E. coli* or *B. subtilis* were added lived the same as *E. coli* fed worms (Figure 3C; Figure S5).

### Dietary coQ from *E. coli* acts as an antioxidant and shortens *C. elegans* life span

**Figure 4:**
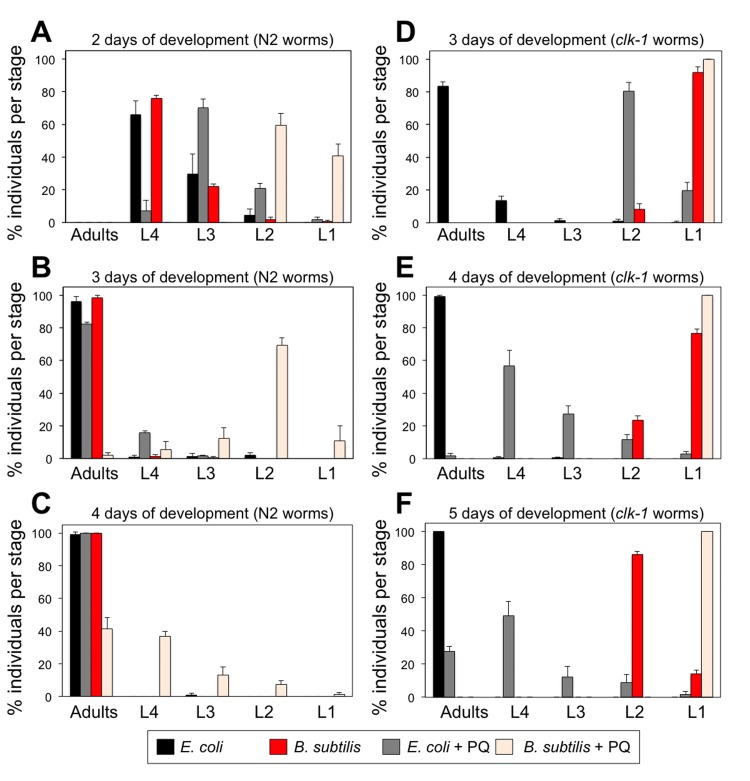
Treatment with PQ severely affects the development of worms feeding on coQ-less *B. subtilis* and the development of coQ-deficient *clk-1* mutant worms (**A**-**C**) Development of synchronized wild type N2 L1 larvae after 2, 3 and 4 days feeding on *E. coli* or *B. subtilis* with or without PQ treatment (0.1 mM). (**A**-**C**) Development of synchronized clk-1(qm30) L1 larvae after 3, 4 and 5 days feeding on E. coli or B. subtilis with or without PQ treatment (0.1 mM). (A-F) y-axis shows percentage of individuals that reached each developmental stage after the indicated time. x-axis shows developmental stages. Bars indicate the mean value ± S.D. n = 118-196 worms per group.

Besides being an essential ETC acceptor/donor of electrons, coQ is also a potent antioxidant [40]. *C. elegans* and *E. coli*, but not *B. subtilis*, synthesize their own coQ. Thus, worms that feed on *E. coli* are subjected to persistent dietary coQ supplementation and this may act as a lifelong antioxidant treatment for the worms affecting negatively their life span. To begin testing this possibility, we maintained WT L1 larvae on *E. coli* or *B. subtilis* seeded plates to which we had added a small concentration of the oxidant paraquat (PQ). If dietary coQ supplementation by *E. coli* indeed acts as an antioxidant, then it should counteract the effect of the oxidant. *E. coli* fed L1 larvae maintained on PQ containing plates suffered a minor developmental delay. However, *B. subtilis* fed L1 larvae maintained on PQ containing plates exhibited a severe developmental delay (Figure 4A-C). We performed the same experiment using coQ-deficient *clk-1* worms. *clk-1* L1 larvae maintained on *E. coli* seeded plates that contained PQ displayed a severe developmental delay compared with control *E. coli* fed *clk-1* L1 larvae (Figure 4D-F). Although *clk-1* L1 larvae are not able to complete development when maintained on *B. subtilis*, the arrested development phenotype was more severe when plates contained PQ (Figure 4D-F). After 14 days, the *B. subtilis* fed *clk-1* L1 larvae had transitioned into sterile adults, whereas all the *B. subtilis* fed *clk-1* larvae maintained on PQ plates continued arrested at L1 stage. The development in the presence of the PQ was also severely affected when WT larvae were fed a coQ-deficient *E. coli* diet (Figure S6). Our results show that worms with more coQ content such as *E. coli* fed WT worms, are able to better offset the oxidative effect of PQ and thus counteract the developmental delay induced by PQ.

**Figure 5:**
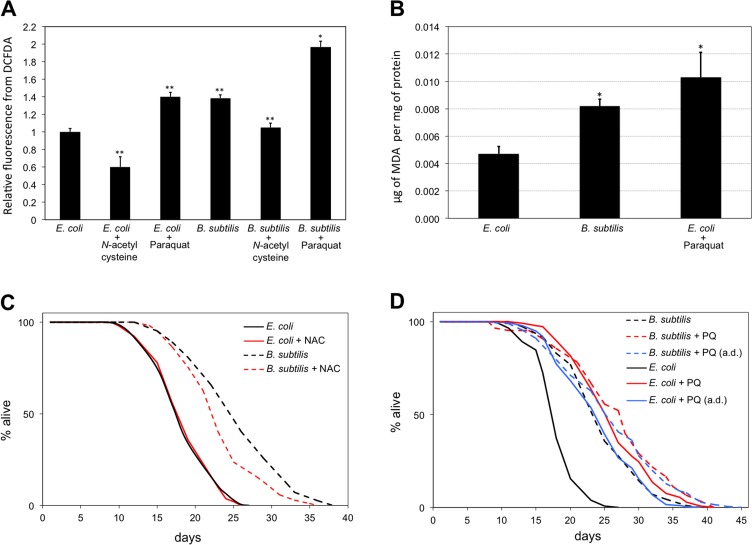
*B. subtilis* fed worms live longer and are in a higher oxidation state than *E. coli* fed worms (**A**) *B. subtilis* fed worms have higher ROS levels than *E. coli* fed worms. Treatment with the antioxidant NAC (10 mM) decreases ROS levels of worms in both diets. Treatment with the pro-oxidant PQ (0.1 mM) increases the ROS levels of worms in both diets. Bars indicate the relative mean fluorescent marker expression ± S.E.M difference to the *E. coli* fed worms control. n = 10-20 for each group (**p < 0.01, *p < 0.05, Student's t test). Statistical significance for *B. subtilis* fed worms treated with NAC and for *B. subtilis* fed worms treated with PQ is established with respect to *B. subtilis* fed worms. y-axis indicates relative fluorescence from DCFDA. x-axis indicates diet and treatment of worms. (**B**) *B. subtilis* fed worms have higher MDA levels than *E. coli* fed worms. n = 3-4 for each group (*p < 0.05, Bonferroni). y-axis indicates μg of MDA per mg of total protein. x-axis indicates diet and treatment of worms. (**C**) Life span curves for adult worms maintained on *E. coli* or *B. subtilis* with or without NAC treatment. (**D**) Life span curves for adult worms maintained on *E. coli* or *B. subtilis* with or without PQ treatment. Worms were subjected to PQ treatment since L1, or since adulthood (a.d.). (**C**-**D**) y-axis indicates percentage of worms that are alive. x-axis indicates day of adulthood. See also Table S5.

If *E. coli* fed worms were indeed subjected to the persistent antioxidant effect induced by coQ supplementation, then they should be in a less oxidized cellular state than *B. subtilis* fed worms. We examined the overall cellular reactive oxygen species (ROS) levels of worms and observed that *B. subtilis* fed worms had cellular ROS levels 40% higher than *E. coli* fed worms (Figure 5A). *E. coli* fed worms maintained on PQ-containing plates also had increased cellular ROS levels and these were similar to those observed for *B. subtilis* fed worms. *E. coli* or *B. subtilis* diet plates that contained the chemical antioxidant N-acetyl-cysteine (NAC) led to worms with approximately 40% lower cellular ROS levels than worms fed solely *E. coli* or *B. subtilis*, respectively (Figure 5A). Worms maintained on *B. subtilis* seeded plates that contained the oxidant PQ displayed higher ROS levels than *B. subtilis* fed worms or *E. coli* fed worms that were maintained on PQ plates (Figure 5A). *E. coli* fed *clk-1* worms as well as coQ-deficient *E. coli* fed WT worms also displayed higher cellular ROS levels than *E. coli* fed WT worms (Figure S7). Next, we used cellular lysates obtained from worms fed either *E. coli* or *B. subtilis* and compared their amounts of malondialdehyde (MDA), a commonly used marker of lipid peroxidation [41]. *B. subtilis* fed worms as well as PQ-treated *E. coli* fed controls had 1.7 and 2.2 fold higher MDA levels than *E. coli* fed worms, respectively (Figure 5B). The ROS and the MDA data indicate that worms feeding on the coQ-containing *E. coli* diet are in a less oxidized cellular state than worms feeding on the coQ-less *B. subtilis* diet.

If the life span difference between *E. coli* and *B. subtilis* fed worms is due to the less oxidized state induced by the persistent coQ antioxidant effect of the *E. coli* diet, then treating *B. subtilis* fed worms with an antioxidant should lead to worms with lower cellular ROS levels and shorter life spans. Treatment of *B. subtilis* fed worms with the antioxidant NAC leads to worms with lower ROS levels than *B. subtilis* fed worms (Figure 5A). NAC treatment had no life span effect on *E. coli* fed worms. However, worms maintained on *B. subtilis* seeded plates that also contained NAC lived shorter than control *B. subtilis* fed worms (Figure 5C). This life span decrease is partial as NAC-treated *B. subtilis* fed worms still lived 27% longer than *E. coli* fed worms. The partial life span reduction effect might be due to other beneficial effects induced by the *B. subtilis* diet such as the lack of LPS.

Treating *E. coli* fed worms with a low concentration of PQ increases cellular ROS levels (Figure 5A) and, as already reported [42,43], worms live longer than untreated *E. coli* fed worms. *B. subtilis* fed worms and PQ-treated *E. coli* fed worms exhibit similar cellular ROS levels (Figure 5A) and live considerably longer than *E. coli* fed worms (Figure 1A; Figure 5A). If the mechanism by which *B. subtilis* fed worms live longer than *E. coli* fed worms is independent of the mechanism by which PQ-treated *E. coli* fed worms live longer than *E. coli* fed worms, then treating *B. subtilis* fed worms with PQ should lead to a further longevity increase. We tested this possibility by measuring the life span of *E. coli* and *B. subtilis* fed worms when these were subjected to PQ treatment. PQ-treated *B. subtilis* fed worms lived only 8.4% longer than PQ-treated *E. coli* fed worms. Moreover, starting the PQ treatment after worms reached adulthood led to a minimal 6.4% life span difference between *E. coli* and *B. subtilis* fed worms (Figure 5D). The partial life span increase observed between PQ-treated *B. subtilis* and PQ-treated *E. coli* fed worms might be due to other beneficial effects induced by the *B. subtilis* diet such as the lack of LPS. Interestingly, coQ-deficient *E. coli* fed worms also failed to further increase their longevity upon PQ treatment (Figure S8). These results indicate that the mechanism by which *B. subtilis*, coQ-deficient *E. coli*, and PQ-treated *E. coli* fed worms live longer than *E. coli* fed worms is likely the same.

### Mutations that lead to life span alterations when worms are fed *E. coli* do not always lead to proportional life span changes when worms are fed *B. subtilis*

Most *C. elegans* aging research is done using *E. coli* as the standard control diet. Since the longevity of *E. coli* fed worms is affected by dietary factors such as the persistent exposure to dietary coQ, we assayed whether genetic interventions that alter longevity by affecting *C. elegans* aging, immunity, and stress response lead to similar results when worms are fed *E. coli* or *B. subtilis*. We used 14 *C. elegans* mutant strains including the insulin/IGF-1-like signaling pathway receptor *daf-2* [44]; two alleles of the *daf-16* forkhead box O (FOXO) transcription factor homologue, *daf-16(mu86)* and *daf-16(mgDf50)*, which regulate insulin/IGF-1-mediated signaling activity [45]; and the double mutant *daf-2;daf-16*. We also used the stress response transcription factor mutant *hsf-1* [46]; and the hypoxia-induced factor mutant *hif-1* [42]. The mutant strains we used that affect the innate immune response were the mitogen-activated protein kinase (MAPK) *pmk-1* [47]; the member of the transforming growth factor beta (TGFβ) superfamily *dbl-1* [48]; the serine/threonine kinase member of the c-Jun N-terminal kinase (JNK) subgroup of mitogen-activated protein kinases *jnk-1* [49]; and the Toll-like receptor (TLR) *tol-1* [50]. We also used several mutant strains that are known to increase worm longevity including the nicotinic acetylcholine receptor subunit *eat-2,* which mimics dietary restriction [51]; the Notch family receptor *glp-1* [52]; and the mitochondrial electron transport chain complex I and III subunits *nuo-6* and *isp-1* [43].

**Figure 6:**
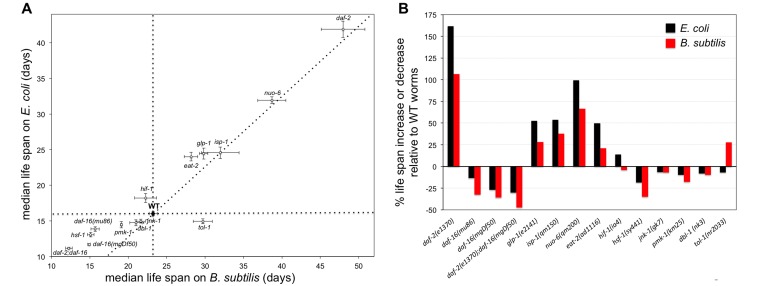
Genetic interventions that affect the life span of worms fed *E. coli* do not always lead to similar effects in worms fed *B. subtilis* (**A**) Median life span ± S.E.M. of 14 mutant strains as well as WT worms when worms are fed *E. coli* or *B. subtilis*. Perpendicular dotted lines are reference lines representing the median life span of WT worms on each diet. The diagonal dotted line represents a perfect proportionality between median life span for mutant worms upon feeding *B. subtilis* relative to feeding *E. coli*, based on the WT median life span results. See also Table S6. (**B**) Bars represent the percentage of life span increase or decrease for mutant worms fed *E. coli* or *B. subtilis* relative to WT worms fed the same type of diet. Indicated is the mutant and allele used in each life span experiment.

WT worms fed *B. subtilis* lived 45% longer than *E. coli* fed WT worms. Using this result as a reference point, we examined the life span difference between the 14 mutants when worms were fed either of the two diets. We observed that in the case of *dbl-1*, *jnk-1*, *isp-1*, *nuo-6*, and *daf-2* mutants the life span difference when worms were fed either diet was similar (Figure 6A and Table S7). However, in the case of the *glp-1*, *eat-2*, *hif-1*, *pmk-1*, *hsf-1*, *daf-16*, *daf-2; daf-16*, and *tol-1* mutants the life span difference with respect to WT worms when worms were fed *E. coli* or *B. subtilis* was not proportionally similar. These mutants could be divided in 4 categories according to their life span differences with respect to WT controls. The two *daf-16* alleles, *daf-2;daf-16*, *pmk-1*, and *hsf-1* mutant worms lived proportionally shorter than WT worms when fed *B. subtilis* than when fed *E. coli* (Figure 6). *glp-1* and *eat-2* mutant worms lived proportionally longer than WT worms when fed *E. coli* than when fed *B. subtilis* (Figure 6). *hif-1* worms displayed a slightly shorter life span than WT worms when fed *B. subtilis*, yet they displayed a slightly longer life span than WT worms when fed *E. coli*. Interestingly, *tol-1* mutant worms showed a strong differential longevity response with respect to WT worms depending on the diet. These worms lived 28% longer than WT worms when fed *B. subtilis*, yet they lived slightly shorter than WT worms when fed *E. coli*. Taken together, the data show that different *C. elegans* signaling pathways involved in aging, immunity and stress response can be more or less relevant for worm survival depending on dietary and microbiome factors.

## DISCUSSION

**Figure 7:**
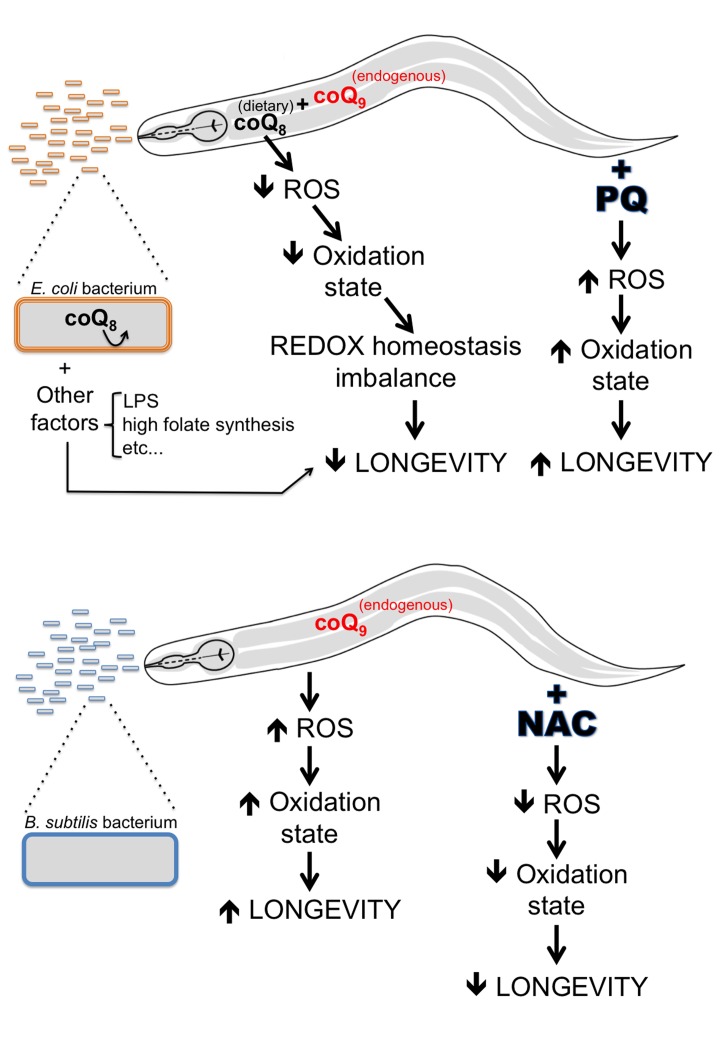
A model explaining life span differences for worms fed *E. coli* vs. *B. subtilis* CoQ-supplemented *E. coli* fed worms have lower ROS levels and a lower oxidation state than coQ-deficient *B. subtilis* fed worms. The lifelong antioxidant effect of coQ supplementation by the *E. coli* diet provokes detrimental alterations in the worm cellular REDOX homeo-stasis, which in turn lead to a decrease of *C. elegans* longevity. Increasing the ROS levels of *E. coli* fed worms by PQ treatment would increase the oxidation state of worms and rebalance the cellular REDOX homeostasis of *E. coli* fed worms. CoQ-deficient *B. subtilis* fed worms have higher ROS levels and a higher oxidation state than coQ-supplemented *E. coli* fed worms. The lack of coQ supplementation by the *B. subtilis* diet leads to worms with a balanced cellular REDOX homeostasis. Decreasing the ROS levels of *B. subtilis* fed worms by NAC treatment would decrease ROS levels and the oxidation state of worms and cause alterations in the cellular REDOX homeostasis of *B. subtilis* fed worms. Other *E. coli* factors (LPS, high folate synthesis, etc.) would also contribute to the life span difference between *E. coli* and *B. subtilis* fed worms

Our work demonstrates that the *C. elegans* life span difference observed when the worm diet is changed from the standard *E. coli* to *B. subtilis* is mainly due to the persistent antioxidant effect of coQ present in the *E. coli* diet. Contrary to *E. coli*, *B. subtilis* do not synthesize or use coQ. Dietary coQ-containing *E. coli* fed worms have lower cellular ROS levels and a less oxidized state than *B. subtilis* fed worms. We propose that this less oxidized cellular state of *E. coli* fed worms leads to an imbalance in cellular REDOX homeostasis, which in turn shortens their life span (Figure 7). According to the REDOX stress hypothesis of aging, altering the worm cellular REDOX homeostasis provokes alterations of ROS-dependent signaling pathways, as well as alterations of enzymatic reactions that are optimized for a particular cellular REDOX state [53]. Our findings highlight the impact that the microbiome can have on longevity, and argue that the excessive use of antioxidants can be detrimental.

Feeding on *B. subtilis* instead of *E. coli* makes worms live longer and lowers their initial mortality rate, suggesting that the *E. coli* diet adds additional risk factors to the worms and induce them to live shorter. Accordingly, it was reported that the *sod-3::GFP* aging biomarker, which predicts remaining life span of synchronous individual worms when fed *E. coli*, is not able to predict the life span of worms fed *B. subtilis* suggesting that these two diets induce different causes of death [19]. In our study we provide several lines of evidence demonstrating that the life span differences of worms fed these two diets are mostly due to detrimental effects induced by *E. coli* feeding. First, worms maintained on *E. coli* during development and then switched to *B. subtilis* for the rest of their lives suffered a longevity shortening with respect to worms that exclusively fed on *B. subtilis*. Second, adding *E. coli* extracts to *B. subtilis* fed worms decreased their longevity, whereas adding *B. subtilis* extracts to *E. coli* fed worms did not have a longevity effect. Third, we showed that feeding worms *B. subtilis* does not result in a state of dietary restriction, which could have explained their longevity increase. We observed that a minor contribution for these life span differences comes from a small beneficial effect caused by the presence of *B. subtilis* spores. The mechanism for the beneficial effect of spores remains to be determined.

Worms fed *B. subtilis* live longer mainly because this food source does not contain coQ. Evidence for this comes from the life span decrease of *B. subtilis* fed worms when a coQ-active *E. coli* extract is added. Yet, when a coQ-deficient *E. coli* extract is added the life span of *B. subtilis* fed worms is minimally affected. Previous studies had shown that commercial supplementation of coQ_10_ (NovaSOL Q) to coQ-deficient *E. coli* fed worms did not decrease their life span [10]. These results led the authors conclude that altered bacterial metabolism, rather than coQ content, is responsible for the lifespan extension of worms fed an *E. coli* diet lacking coQ. However, it is worth noting that potential issues exist when using this type of commercial coQ. For example, the NovaSOL vehicle is necessary to deliver the highly hydrophobic coQ_10_ into the aqueous *C. elegans* media [10]. Using this method, even when only the NovaSOL vehicle was added to worms fed coQ-deficient *E. coli* or to worms fed standard *E. coli*, an increase in longevity by 20% and 16% was achieved, respectively [10]. Therefore, the vehicle in which coQ_10_ is administered might mask the longevity effects of NovaSOL coQ_10_ supplementation. In fact, NovaSOL coQ_10_-supplemented *E. coli* fed worms lived shorter than NovaSOL vehicle-only supplemented *E. coli* fed worms [10].

Our alternative coQ supplementation approach ensured effective coQ delivery to the worm as well as supplementation with the same coQ species as worms obtain when fed the laboratory *E. coli* diet. Supplementing the *B. subtilis* diet with *E. coli* extract made worms live shorter than worms solely fed *B. subtilis.* However, when the *B. subtilis* diet was supplemented with a coQ-deficient *E. coli* extract, worm longevity was minimally affected. We concluded that *B. subtilis* fed worms live longer than *E. coli* fed worms mainly because this food source does not contain coQ. The small life span decrease of *B. subtilis* fed worms in the presence of coQ-deficient *E. coli* extract could be explained by the negative impact of other *E. coli* factors still present in the extract such as LPS or folic acid. *E. coli* infection also partially contributes to the life span differences between *E. coli* and *B. subtilis* fed worms as feeding worms UV killed instead of live *E. coli* increases worm life span by 20%. The role that *E. coli* infection plays in the shortening of life span could be due to a dysbiosis phenomenon caused by excessive bacterial gut proliferation [11].

Saiki et al. have suggested that metabolic differences derived from coQ-active and deficient bacteria diets lead to worm longevity differences [10]. Our data lead us to propose a different model. Besides playing an essential role in the ETC, coQ is also a potent antioxidant [40]. *E. coli* fed worms are subjected to persistent dietary coQ supplementation, which acts as a lifelong antioxidant treatment and shortens *C. elegans* life span by altering the worm cellular REDOX homeostasis. Evidence that *E. coli* fed worms have a less oxidized state than *B. subtilis* fed worms due to dietary coQ supplementation, and that this less oxidized state leads to a worm longevity decrease comes from four corroborative results. First, coQ-containing *E. coli* fed worms have lower cellular ROS levels and lower cellular lipid peroxidation levels than coQ-less *B. subtilis* worms. Second, development of the more oxidized *B. subtilis* fed larvae is severely affected by further oxidation upon PQ treatment, whereas development of the less oxidized *E. coli* fed larvae is minimally affected upon PQ treatment. Third, treating *B. subtilis* fed worms with the antioxidant NAC decreased both cellular ROS levels as well as longevity. Fourth, treating the less oxidized *E. coli* fed worms with PQ increased, as previously reported [42,43], worm cellular ROS levels as well as longevity. However, treating the already more oxidized *B. subtilis* fed worms with PQ only led to a minor longevity increase.

We propose that coQ supplementation from the *E. coli* diet acts as lifelong antioxidant treatment and shortens *C. elegans* life span by altering the cellular REDOX homeostasis of the worm. The cellular REDOX state has been shown to play essential roles in gene regulation, cell signaling, differentiation, apoptosis, etc. This has led to the proposal of the REDOX stress hypothesis of aging [53]. Therefore, a lower cellular oxidation state induced by persistent coQ supplementation would disturb REDOX-sensitive signaling mechanisms in a systemic way and thus negatively influence longevity.

Despite living longer, *B. subtilis* fed worms have higher ROS levels than *E. coli* fed worms. An often-prevalent claim is that keeping cellular ROS levels low by using antioxidant treatments will reduce oxidative stress and consequently be beneficial for organismal health and survival [54]. Our data do not support this view but rather agree with recent reports that question the beneficial use of antioxidants [55,56]. For example, a recent study showed that lifelong antioxidant treatment accelerated mice lung cancer progression [56]. Additionally, a large human epidemiological review revealed that antioxidant supplements are not associated with lower all-cause mortality and that, at higher doses, certain types of antioxidant supplements may be associated with higher all-cause mortality [55]. Our findings posit that antioxidant supplementation affects cellular REDOX balance and alter proper cellular function. Thus, persistent antioxidant supplementation in humans might cause similar effects as those reported here for *C. elegans*.

*E. coli* is a convenient laboratory diet for *C. elegans*. However, the longevity of *E. coli* fed worms is affected by dietary coQ supplementation as well as by other factors like LPS, which are not present in *B. subtilis*. Thus, feeding *E. coli* to worms can lead to misestimating the effect that a given signaling pathway can have on longevity. In fact, we report that mutant *C. elegans* strains that affect aging, the immune system, or stress response behave differently depending on whether worms are fed *E. coli* or *B. subtilis*. For example, *eat-2* mutant worms, which mimic dietary restriction, lived 50% longer than WT worms when fed *E. coli*. However, when *B. subtilis* was used as diet, *eat-2* worms lived only 21% longer than WT worms. The *eat-2* mutation increases longevity but it appears that this mutation also confers protection against the detrimental effects of *E. coli*, which is translated in an additional longevity increase with respect to WT worms when these are fed *E. coli*.

From all the tested mutants, *hif-1* worms were the only ones that lived longer than WT worms when fed *E. coli*, but shorter when fed *B. subtilis*. This suggests that longevity of *B. subtilis* fed worms is partially dependent on HIF-1 activity. Lee et al. reported that inhibition of respiration extends *C. elegans* life span via ROS that increase HIF-1 activity. They also reported that the longevity increase of *E. coli* fed worms upon PQ treatment, which induces elevated ROS levels, was partially dependent on HIF-1 activity [42]. We speculate that the longevity of *B. subtilis* fed worms is also partially dependent on HIF-1, which in turn is activated by the higher ROS levels of *B. subtilis* fed worms.

*tol-1* worms were the only mutants that lived longer than WT worms when fed *B. subtilis*, yet they lived slightly shorter than WT worms when fed *E. coli*. TOL-1 is a Toll-like receptor and has been shown to be required for the worm innate immune response [50]. It was shown that the survival of *tol-1* worms is significantly more affected than the survival of WT worms when these are in the presence of the gram negative *E. coli* or *Salmonella enterica*. However, the survival of *tol-1* worms in the presence of the gram positive pathogen *Enterococcus faecalis* is considerably longer than that of WT worms [50]. According to these authors and in agreement with our own results, TOL-1 has a direct role in the immune response to certain gram negative bacteria. It was reported that worms fed the gram positive *Bifidobacterium infantis* lived longer than *E. coli* fed worms, and that *tol-1* mutant worms lived longer on *B. infantis* than WT worms [57]. We speculate that TOL-1 functions to protect *C. elegans* against gram negative specific factors, but that the activity of this protection system might be detrimental as when TOL-1 is not functional and the gram negative threat is not present (worms are fed gram positive bacteria), worms live longer than WT worms. Yet, if the gram negative threat is present and TOL-1 is not functional, then worm survival is worsened with respect to WT worms.

Our study shows that the longevity difference when feeding *B. subtilis* to *C. elegans* instead of *E. coli* is not a mere consequence of one diet being more nutritious than the other, but rather due to one diet containing factors that are detrimental to the worm. We also showed that signaling pathways that affect longevity can have more or less of an impact depending on the diet worms are fed. Our study illustrates the importance that the microbiome can have on influencing life expectancy.

## MATERIALS AND METHODS

### Reagents

NAC (Sigma) was prepared as 500 mM stock solution in distilled water. PQ (Sigma) was prepared as 1 M stock solution in distilled water. FUDR (Sigma) was prepared as 30 mM stock solution in distilled water. H_2_DCFDA (Life Technologies) was prepared as 10 mg/ml stock solution in DMSO and stored at −20°C. MDA standard was purchased from Sigma.

### *C. elegans* maintenance

Worms were maintained and handled as previously described [2]. Unless otherwise stated, all the reagents used in *C. elegans* experiments were added to the molten NGM agar and cast into culture plates. Hardened agar was seeded with the proper bacterial culture. All *C. elegans* experiments were conducted at 20°C.

### *C. elegans* development assay

*C. elegans* embryos were isolated following standard bleaching protocol using a 1:2 solution of 5 M NaOH:5% NaOCl [2]. Embryos were allowed to hatch overnight at 20°C in M9 buffer. Arrested L1 larvae were transferred to NGM plates seeded with the proper bacterial culture. Worms were examined over time and the percentage of adults and larvae at different stages was determined.

### *C. elegans* progeny determination

Total progeny number was determined as previously published [58] by counting the daily as well as the cumulative number of progeny from 5 single worms.

### Dietary restriction conditions

We subjected adult worms to solid dietary restriction (sDR) as previously described [59] with slight modifications. sDR conditions were achieved by seeding plates with 10^8^ CFU/ml (strong sDR) or with 10^9^ CFU/ml (mild sDR).

### *C. elegans* lifespan assay

Synchronized L4 larvae were transferred to bacterial seeded NGM plates containing FUDR (15 μM). 200 μl of the corresponding bacterial culture were used to seed plates. When a specific bacterium was used for a life span experiment, worms were fed this type of bacteria during development and adulthood. Life span assays with UV-killed *E. coli* or *B. subtilis* were done by UV treating bacteria as described [24]. Plates were scored for dead worms every other day. Worms that did not respond to prodding with a platinum wire were considered dead. Age refers to days following adulthood. Individuals were excluded from the analysis when their gonad was extruded, or when they desiccated by crawling onto the edge of the housing plate.

### *C. elegans* fluorescence microscopy

A Zeiss Axioplan microscope equipped with Zeiss AxioVision 4.6 software and a Leica DM6000B microscope equipped with Metamorph 7.6.2.0 software were used for quantitative fluorescence microscopy. Worm gut autofluorescence was imaged using a 650 nm bandpass filter. Images were captured with 20X lens and analyzed using ImageJ. To analyze fluorescence, the same area size was selected from worm to worm. This area included the anterior part of the intestine. Gut autofluorescence time courses were done using 10-15 worms per age. All pictures were taken on the same day with the same microscope settings. *unc-54::mCherry* and *pha-4::mCherry* fluorescence worms were photographed using 10x or 20x lenses and images were analyzed using ImageJ. All the *mCherry* fluorescence experiments were done using at least 15 worms per treatment and/or per age. For a given experiment, all pictures were taken on the same day with the same microscope settings. To analyze *unc-54::mCherry* and *pha-4::mCherry* fluorescence, the same area size was selected from worm to worm. In the case of *unc-54::mCherry* worms this area included the head of the worm until the end of the pharynx. In the case *pha-4::mCherry* worms this area included the first two pairs of intestinal cells.

### ROS determination

2:1 parts of M9 buffer:H_2_DCFDA stock solution were mixed and divided into 0.5 ml tubes. 2-day-old worms of each sample were placed in each tube and incubated for 20 minutes at room temperature with moderate shaking (protected from the light). Worms were then centrifuged at 2000 rpm and washed twice with M9. Worms were pipetted onto agar pads (2% agarose in water) that were prepared on glass slides and covered with a coverslip. Individual worms were photographed using the GFP channel at 10X magnification. Fluorescence intensity was analyzed with ImageJ. To analyze fluorescence, the same area size was selected from worm to worm. This area included the head and the first two pairs of intestinal cells.

### Bacterial extract preparations

40 ml of fresh overnight *E. coli* or *B. subtilis* cultures were distributed into conical tubes and incubated at 70°C in a water bath for 30 minutes. 0.4 ml of WEC lysis buffer was added to each of the 40 ml samples and these were incubated at 4°C for 30 minutes. Samples were then subjected to 4 cycles of liquid nitrogen followed by 30°C water bath and centrifuged (4000 rpm, 15 minutes, 4°C). After centrifugation, 28 ml of supernatant were discarded from each 40 ml tube. The pellet extract was then resuspended in the remaining 12 ml of supernatant. Dilutions (dH_2_O) of the extract (1:2, 1:5, 1:10, 1:20) were done to prepare the extract at different concentrations. Prior to using an extract, we verified that there were not bacteria alive by inoculating two LB plates with 200 μl of extract and incubating them overnight at 37°C. *E. coli* (GD1) and *B. subtilis* bacterial cultures grew at a concentration of fewer CFU/ml than *E. coli* (OP50). Thus, bacterial extracts were concentrated to achieve CFU/ml equivalent to those obtained for *E. coli* (OP50). 75 μl of extracts were dispensed on top of the bacterial lawn of worm plates every other day for the first 20 days of worm adulthood as well as during development.

### Bacterial nutritional composition

To determine the water content, approximately 5 g of pelleted bacteria were placed in conical tubes and samples were freeze-dried. The water content was determined from the loss of weight of the samples before and after lyophilization.

Protein composition was determined by the Kjeldalh method (AOAC International, 2005a). Bacterial pellets were freeze-dried and 0.2-0.3 g of each sample were used in the procedure. Samples were transferred into a Kjeldahl digestion flask containing 10 g of catalyst (96% K_2_SO_4_/4% CuSO_4_.5H_2_O) and 20 ml of H_2_SO_4_ and subjected to 2 hours of digestion in a Kjeldalh digestion stand. Once the samples were cooled down at room temperature, the minerals were transferred to a distillation apparatus with 400 ml of distilled water, phenolphthalein as indicator, and NaOH (30%). The ammonia gas liberated from the solution was transferred from the digestion flask into the receiving flask, which contained an excess of H_2_SO_4_ (25 ml, 0.05385 M). The excess of H_2_SO_4_ was titrated by a NaOH solution (0.1093 M) using a mixed indicator endpoint (0.1 g/100 ml bromocresol green and 0.1 g/100 ml methyl red in 95% ethanol). The nitrogen content was determined and protein content was calculated using the conversion factor 6.25.

Fat composition was determined by the Soxhlet method (AOAC International, 2005b). Crude fat content was determined by extracting the fat from the sample using diethyl ether as solvent, and then by weighing the fat that was recovered. Bacterial pellets were freeze-dried and 0.8-1.2 g of each sample were used in the procedure. Samples were introduced in an extraction thimble, which was placed in a Soxhlet apparatus. Approximately 300 ml of diethyl ether were added. The Soxhlet apparatus was placed in a water bath (40°C) prior to the extraction. The solvent completed 20 cycles of extraction, then it was removed and the fat extract was placed in a desiccation stove (100°C) to remove the ether and to allow samples to dry completely. The fat content was calculated from the difference in weights before and after the procedure.

To determine the ash composition, bacterial pellets were freeze-dried and 1.5 g of each sample were placed in incineration melting pots (previously heated at 910°C for 15 min), allowed to cool in a desiccator, and weighed as soon as they reached room temperature. Samples were placed in incineration melting pots in a uniform layer and placed at the entrance of the oven with the door open. Incineration was kept at 910°C until total combustion of the samples. Incineration melting pots were removed from the oven, allowed to cool in a desiccator and weighed as soon as they reached room temperature. Ash content was calculated from the weight difference before and after incineration. Carbohydrate composition was determined by the difference between the total weight of the dehydrated sample minus protein, fat and ashes.

### Determination of MDA by dinitrophenylhydrazine assay

MDA determination was based on HPLC measurement after derivatization with 2,4-dinitrophenylhydrazine (DNPH) as described [60]. 500 μl of trichloroacetic acid was added to 400 μl of worm homogenate to remove proteins. After 15 min at 4°C the homogenate was centrifuged (10000 g for 10 min at 4°C) and the supernatant was collected and mixed with 100 μl of 5 mM DNPH in 2M HCl (prepared daily) and incubated for 1 hour at room temperature. The mixture was extracted three times with 400 μl of chloroform. The organic phase was collected and molecular sieves were added to the organic phase for 30 min. The molecular sieves were removed and the organic phase concentrated to dryness under nitrogen steam, and resuspended in 80 μl of acetic acid:acetonitrile (62:38, v/v), sonicated twice for 5 s, centrifuged (10000 g, 5 min) and injected in the HPLC system. A calibration curve with concentrations ranging from 0.25 to 100 μg/ml of MDA standard was performed. Protein content in worm homogenate was determined by the Bradford method [61] after digestion of the homogenate.

### HPLC-DAD-ESI/MS analysis

We used a Hewlett-Packard 1100 chromatograph (Agilent Technologies) with a quaternary pump and a diode array detector (DAD) coupled to an HP Chem Station (rev. A.05.04) data-processing station. The column was a Waters Spherisorb S3 ODS-2 C8, 3 μm (4.6 × 150 mm) and the solvents were: (A) 0.2% acetic acid, and (B) acetonitrile. The elution gradient established was: isocratic 38% of B for 10 min, from 38% to 75% of B in 10 min, from 75% to 76% of B in 4 min, from 76% to 77% in 11 min, from 77% to 80% B in 5 min and wash and re-equilibrate the column. The flow rate established was 0.6 ml/min. Double online detection was carried out in the DAD using 310 nm, 360 nm and 380 nm as preferred wavelengths and in a mass spectrometer connected to HPLC system via the DAD cell outlet. Ms detection was performed in an API 3200 Qtrap (Applied Biosystems) equipped with an APCI source and a triple quadrupole-ion trap mass analyzer, which was controlled by the Analyst 5.1 software. The analytes were ionized in negative ion mode. The APCI temperature was set at 400°C, the nebulizer gas was 40 psi. Nitrogen served as the curtain was 10 psi and collision gas (medium). Data acquisition was performed in Enhanced Ms analysis mode, which produces single Ms type spectra. Method settings were: declustering potential (DP), −20 V; entrance potential (EP), −10 V; and collision energy (CE), −10V. In order to obtain the fragmentation pattern of the parent ion, enhanced product ion (EPI) mode was also applied using the following settings: declustering potential (DP), −40 V; entrance potential (EP), −6 V and collision energy (CE), −10V. MDA was quantified from their chromatographic peak recorded at 310 nm compared with calibration curve of the standard.

## SUPPLEMENTAL DATA FIGURES AND TABLES



## Abbreviations

coQ: coenzyme Q
ETC: electron transport chain
LPS: lipopolysaccharides
MDA: malondialdehyde
NAC: N-acetyl-cysteine
PQ: paraquat
ROS: reactive oxygen species
