# Supplementary material for: Dietary and microbiome factors determine longevity in Caenorhabditis elegans
Source: Aging (Albany NY). 2016 Jul 31;8(7):1513–30. doi: 10.18632/aging.101008 (PMC4993345; doi:10.18632/aging.101008)
Supplement: Supplementary file 1 [file aging-08-1513-s001.pdf]

SUPPLEMENTAL DATA

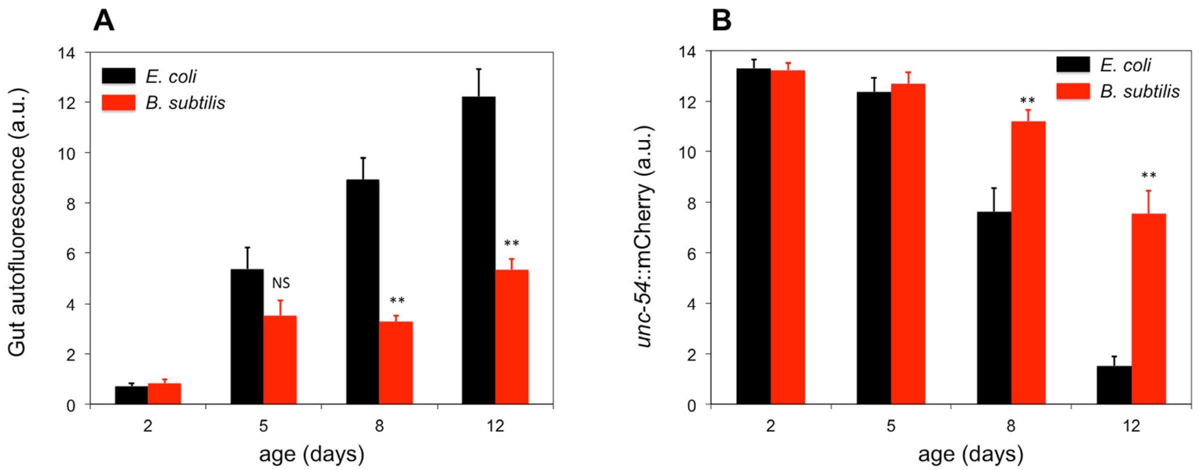

Figure S1. Gut autofluorescence and *unc-54::mCherry* reporter as indicators of the overall health of *B. subtilis* and *E. coli* fed worms. (**A**) Gut autofluorescence intensity for *B. subtilis* and *E. coli* fed adult hermaphrodites during aging. y-axis shows levels of gut autofluorescence in arbitrary units. x-axis shows age of worms. (**B**) *unc-54::H1::mCherry* fluorescent marker expression for *B. subtilis* and *E. coli* fed adult hermaphrodites during aging. y-axis shows levels of fluorescent expression in arbitrary units. x-axis shows age of worms. (A-B) Bars indicate the mean fluorescent marker expression ± S.E.M. n = 10-15 for each group (**p < 0.01; NS p > 0.05; Student's t test).

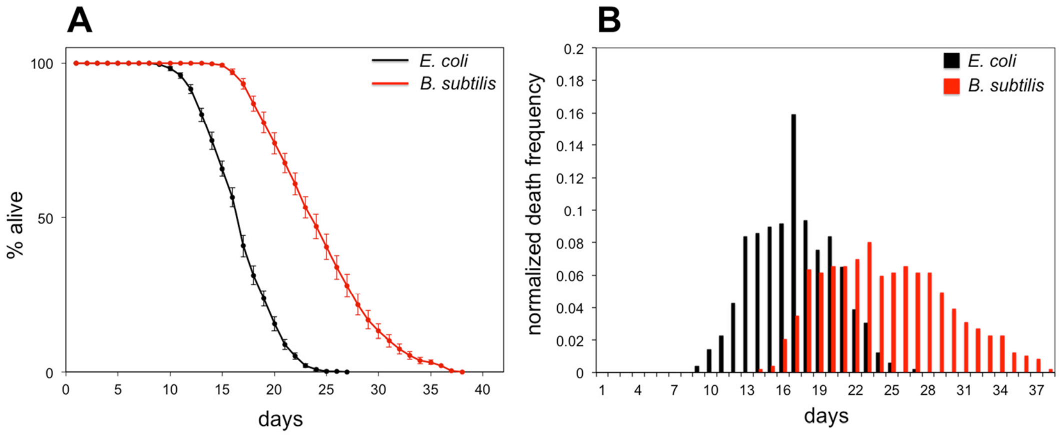

	E. coli (OP50)	*B. subtilis* (PY79)
Number of worms	491	488
Median life span (±SD)	16.4±0.6	23.7±1.7
Life span difference	44.5%	
P. value (log rank)	<0.0001	

Figure S2. ***B. subtilis* fed worms live longer than *E. coli* fed worms and display different demographics of death**. (**A**) Represented are the life span curves for adult hermaphrodite worms maintained on *E. coli* (OP50) or on *B. subtilis* (PY79). The number of dead worms was counted on a daily basis. The worm populations were divided into 10 plates. The points in the life span curves represent the daily mean percentage of alive worms in the 10 plates ± S.E.M. y-axis indicates percentage of worms that are alive. x-axis indicates day of adulthood. (**B**) Represented are the daily death frequencies normalized to their respective sample size.

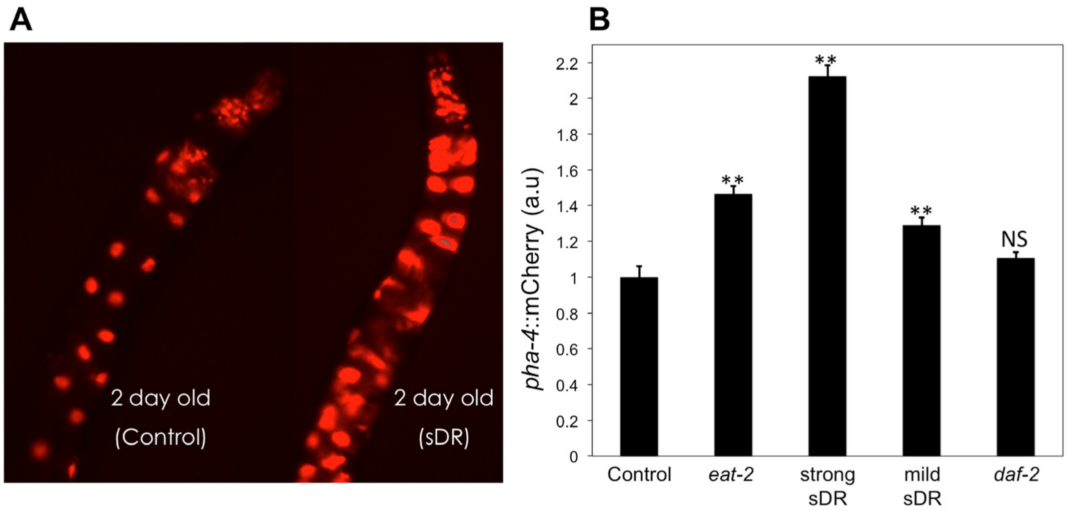

Figure S3. *pha-4::mCherry* reporter as a worm nutritional indicator. (**A**) Representative pictures of *pha-4::H1::mCherry* fluorescent marker expression for 2 day old WT adult hermaphrodites, which were exposed to plenty of food (Control) or dietary restricted (sDR). (**B**) *pha-4::H1::mCherry* fluorescent marker expression for 2 day old WT adult hermaphrodites, which were exposed to plenty of food (Control) or dietary restricted (sDR); and for 2 day old *eat-2(ad1116)* and *daf-2(e1370)* mutant adult hermaphrodites, which were exposed to plenty of food. For details on dietary restriction conditions see the Materials and Methods section. y-axis shows levels of fluorescent expression in arbitrary units. x-axis shows the type of worms and the diet condition. Bars indicate the mean fluorescent marker expression ± S.E.M. n = 15 for each group (**p < 0.01, Student's t test). (**A**-**B**) In all cases worms were fed *E. coli*.

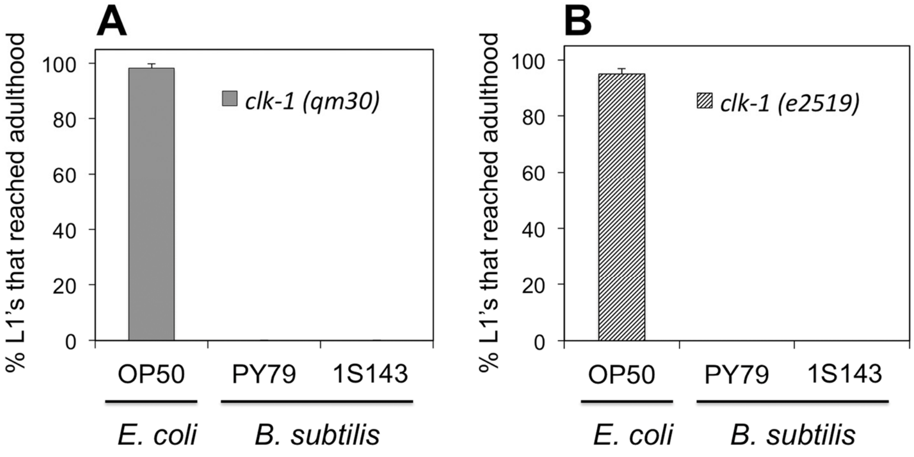

Figure S4. ***clk-1* mutant worms are unable to develop into adults when fed the *B. subtilis* diet**. (**A**) Percentage of *clk-1(qm30)* L1 larvae that developed into adults after 3.9 days of feeding on *E. coli* (OP50), *B. subtilis* (PY79) or the spore-less *B. subtilis* (1S143). (**B**) Percentage of *clk-1(e2519)* L1 larvae that developed into adults after 4.0 days of feeding on *E. coli* (OP50), *B. subtilis* (PY79) or the spore-less *B. subtilis* (1S143). (**A**-**B**) y-axis shows percentage of L1 larvae that reached adulthood. x-axis shows the diet used. Bars indicate the mean value ± S.E.M. n = 140-160 worms per group.

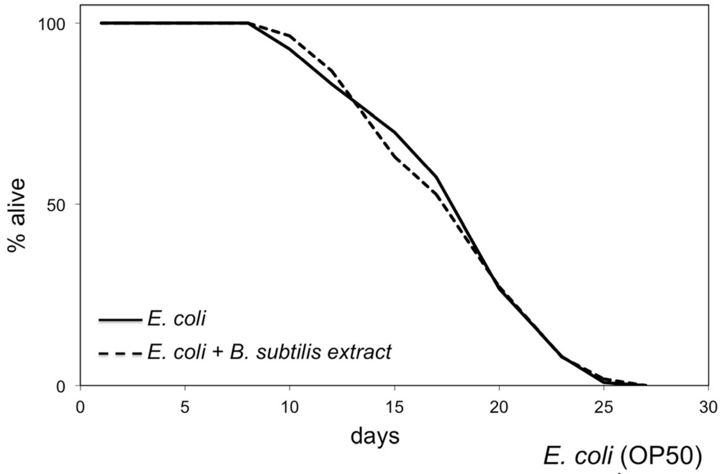

	E. coli (OP50)	*E. coli* (OP50) + *B. subtilis extract*
Number of worms	113	114
Median life span (±SD)	17.4±0.6	17.1±1.1
Life span difference	-1.4%	
P. value (log rank)	>0.05	

Figure S5. *E. coli* fed worms supplemented with coQ-less *B. subtilis* extract do not alter their longevity. Represented are the life span curves for adult hermaphrodite worms maintained on *E. coli* with or without supplementation of coQ-less *B. subtilis* extract. The spore-less *B. subtilis* (1S143) strain was used to prepare the *B. subtilis* extract with the purpose of avoiding the presence of *B. subtilis* spores in the extract. y-axis indicates percentage of worms that are alive. x-axis indicates day of adulthood.

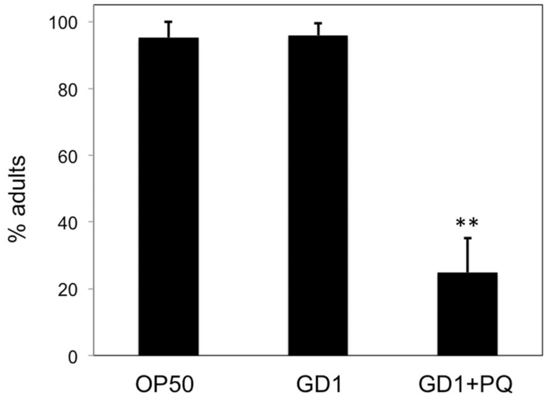

Figure S6. Treatment with a mild dose of paraquat severely affects the development of worms feeding on coQ-defective *E. coli* (GD1) bacteria. Development of synchronized wild type N2 L1 larvae after 3 days feeding on *E. coli* OP50 and *E. coli* GD1 with or without PQ treatment (0.1 mM). y-axis shows percentage of individuals that reached adulthood after 3 days. x-axis shows the type of *E. coli* and the treatment. Bars indicate the mean value ± S.D. n = 114-127 worms per group. **$p < 0.001$, Student's t test.

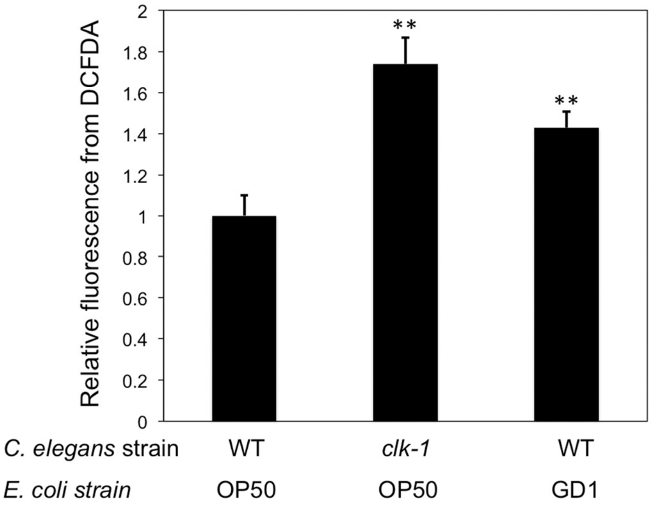

Figure S7. ***E. coli*** **(GD1) fed wild type worms and** ***E. coli*** **(OP50) fed** ***clk-1(qm30)*** **worms have higher ROS levels than** ***E. coli*** **fed WT worms.** Bars indicate the relative mean fluorescent marker expression ± S.E.M difference relative to the *E. coli* fed WT worms. n = 14-20 for each group (**p < 0.01, Student's t test). y-axis indicates relative fluorescence from DCFDA. x-axis indicates diet and type of worms.

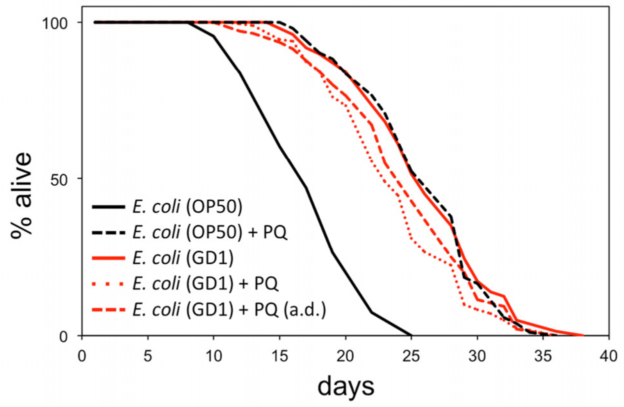

	OP50	OP50+PQ	GD1	GD1+PQ	GD1+PQ(a.d.)
Number of worms	68	103	208	140	184
Median life span (±SD)	16.5±1.0	25.5±1.1	25.2±1.1	22.9±0.9	23.8±1.8
Life span difference		54.5%	52.7%	38.8%	44.2%
P. Value (log rank)		< 0.0001	< 0.0001	< 0.0001	< 0.0001

Figure S8. Life span extension of paraquat-treated worms depends on the presence of coQ in the *E. coli* diet. Represented are the life span curves for adult worms maintained on coQ-active *E. coli* OP50 or coQ-deficient *E. coli* GD1 with or without PQ treatment (0.1 mM). Worms were subjected to PQ treatment since L1, or since adulthood (a.d.). y-axis indicates percentage of worms that are alive. x-axis indicates day of adulthood.

Table S1. Supporting life span data for Figure 1

C. elegans strain	Bacterial food strain	Sample size	Median life span ± SD	% Life span change	P. value (log rank)
Wild type (N2)	*E. coli* (OP50)	178	14.2±0.8		
Wild type (N2)	*B. subtilis* (168)	169	20.4±0.5	+43.1%	<0.0001
Wild type (N2)	*B. subtilis* (3610)	148	22.2±1.5	+56.0%	<0.0001
Wild type (N2)	*B. subtilis* (PY79)	170	22.6±1.0	+58.5%	<0.0001

C. elegans strain	Bacterial food strain	Sample size	Median life span ± SD	% Life span change	P. value (log rank)
Wild type (N2)	*E. coli* (OP50)	107	15.5±0.3		
Wild type (N2)	*B. subtilis* (PY79)	118	25.2±0.4	+62.6%	<0.0001
Wild type (N2)	*B. subtilis* (1S143)	94	22.7±1.2	+46.5%	<0.0001

C. elegans strain	Bacterial food strain	Sample size	Median life span ± SD	% Life span change	P. value (log rank)
Wild type (N2)	*E. coli* (OP50)	76	15.9±0.4		
Wild type (N2)	*B. subtilis* (PY79)	132	23.0±0.7	+44.7%	<0.0001
Wild type (N2)	UV killed *B. subtilis* (PY79)	81	22.7±0.8	+42.8%	<0.0001
Wild type (N2)	UV killed *E. coli* (OP50)	96	19.2±0.4	+20.8%	<0.001

C. elegans strain	Bacterial food strain	Sample size	Median life span ± SD	% Life span change	P. value (log rank)
Wild type (N2)	*E. coli* (OP50)	107	15.5±0.3		
Wild type (N2)	*B. subtilis* (PY79)	118	25.2±0.4	+62.6%	<0.0001
Wild type (N2)	Worms developed from L1 to late L4 on *E. coli* (OP50), then switched to *B. subtilis* (PY79)	111	19.3±2.4	+24.5%	<0.0001

Table S2. Nutritional composition of *E. coli vs. B. subtilis*

	E. coli (OP50)	*B. subtilis* (PY79)
% Water content	78.02	78.75
Protein*	84.72	77.04
Fat*	0.41	0.28
Carbohydrate*	4.51	9.79
Ashes*	10.37	12.89
Calories**	360.57	349.82

* Grams per 100 g of dry bacteria

** Calories per 100 g of dry bacteria

Table S3. Development of *clk-1* mutant L1 larvae after 7 days on the *B. subtilis* diet with *E. coli* extract supplementation

C. elegans strain	Bacterial food strain	Extract supplemented*	Extract dilution	Outcome upon extract supplementation
clk-1(qm30)	*B. subtilis* (PY79)	*E. coli* (OP50)	Undiluted	L1s developed into adults and these produced progeny that arrested at L1-L2 stages
clk-1(qm30)	*B. subtilis* (PY79)	*E. coli* (OP50)	1:2	L1s developed into adults and some of these produced progeny that arrested at L1-L2 stages
clk-1(qm30)	*B. subtilis* (PY79)	*E. coli* (OP50)	1:5	L1s developed into adults and these laid dead embryos
clk-1(qm30)	*B. subtilis* (PY79)	*E. coli* (OP50)	1:10	Some L1s developed into adults
clk-1(qm30)	*B. subtilis* (PY79)	*E. coli* (OP50)	1:20	Most L1s developed into L4s

* single dose of *E. coli* extract was used

Table S4. Supporting life span data for Figure 3

C. elegans strain	Bacterial food strain	Sample size	Median life span ± SD	% Life span change	P. value (log rank)
Wild type (N2)	*E. coli* (OP50)	236	16.4±1.1		
Wild type (N2)	*E. coli* (OP50) + *E. coli* (OP50) extract	118	17.3±0.4	+5.5%	>0.05
Wild type (N2)	*E. coli* (OP50) + *E. coli* (OP50) extract (1:5 dilution)	188	16.7±0.8	+1.8%	>0.05
Wild type (N2)	*B. subtilis* (PY79)	247	24.0±1.0	+46.3%	<0.0001
Wild type (N2)	*B. subtilis* (PY79) + *E. coli* (OP50) extract	117	18.4±1.0	+12.2%	<0.005
Wild type (N2)	*B. subtilis* (PY79) + *E. coli* (OP50) extract (1:5 dilution)	187	20.6±1.2	+25.6%	<0.0001
Wild type (N2)	*B. subtilis* (PY79) + *E. coli* (OP50) extract (1:10 dilution)	79	20.7±1.4	+26.2%	<0.0001
Wild type (N2)	*E. coli* (OP50)	120	16.4±0.8		
Wild type (N2)	*E. coli* (OP50) + *E. coli* (GD1) extract	84	16.7±0.3	+1.8%	>0.05
Wild type (N2)	*E. coli* (OP50) + *E. coli* (GD1) extract (1:5 dilution)	108	16.4±0.4	+0.2%	>0.05
Wild type (N2)	*B. subtilis* (PY79)	92	24.1±0.9	+47.0%	<0.0001

Wild type (N2)	*B. subtilis* (PY79)<br>+<br>*E. coli* (GD1) extract	80	22.4±0.5	+36.6%	<0.0001
Wild type (N2)	*B. subtilis* (PY79)<br>+<br>*E. coli* (GD1) extract (1:5 dilution)	113	24.1±0.6	+47.0%	<0.0001
Wild type (N2)	*B. subtilis* (PY79)<br>+<br>*E. coli* (GD1) extract (1:10 dilution)	122	24.1±1.0	+47.0%	<0.0001

Table S5. Supporting life span data for Figure 5

***C. elegans* strain**	**Bacterial food strain**	**Sample size**	**Median life span ± SD**	**% Life span change**	**P. value (log rank)**
Wild type (N2)	*E. coli* (OP50)	202	17.4±0.5		
Wild type (N2)	*E. coli* (OP50)<br>+ 10mM NAC	195	17.5±1.0	+0.6%	>0.05
Wild type (N2)	*B. subtilis* (PY79)	235	24.5±0.6	+40.8%	<0.0001
Wild type (N2)	*B. subtilis* (PY79)<br>+ 10 mM NAC	102	22.1±1.0	+27.0%	<0.001

***C. elegans* strain**	**Bacterial food strain**	**Sample size**	**Median life span ± SD**	**% Life span change**	**P. value (log rank)**
Wild type (N2)	*E. coli* (OP50)	150	17.2±0.6		
Wild type (N2)	*E. coli* (OP50)<br>+ 0.1 mM PQ	146	25.1±1.5	+45.9%	<0.0001
Wild type (N2)	*E. coli* (OP50)<br>+ 0.1 mM PQ (after development)	180	23.6±1.6	+37.2%	<0.0001
Wild type (N2)	*B. subtilis* (PY79)	169	23.2±1.2	+34.9%	<0.0001
Wild type (N2)	*B. subtilis* (PY79)<br>+ 0.1 mM PQ	151	27.2±2.0	+58.1%	<0.0001
Wild type (N2)	*B. subtilis* (PY79)<br>+ 0.1 mM PQ (after development)	180	25.1±1.8	+45.9%	<0.0001

Table S6. Supporting life span data for Figure 6

***C. elegans* strain**	**Molecular pathway or process affected**	**Bacterial food strain**	**Sample size**	**Median life span ± SEM**	**Bacterial food strain**	**Sample size**	**Median life span ± SEM**	**% Life span change**	**P. value (log rank)**
Wild type (N2)		*E. coli* (OP50)	512	16.0±0.3	*B. subtilis* (PY79)	465	23.2±0.5	+45.1%	<0.0001
daf-2(e1370)	Insulin-like pathway	*E. coli* (OP50)	159	41.9±1.1	*B. subtilis* (PY79)	90	48.0±2.8	+14.5%	<0.001
daf-16(mu86)	Insulin-like pathway	*E. coli* (OP50)	346	13.9±0.3	*B. subtilis* (PY79)	229	15.7±0.5	+13.2%	<0.0005
daf-16(mgDf50)	Insulin-like pathway	*E. coli* (OP50)	117	11.7±0.1	*B. subtilis* (PY79)	115	14.9±0.2	+27.4%	<0.0001
daf-2(e1370); daf-16(mgDf50)	Insulin-like pathway	*E. coli* (OP50)	107	11.2±0.1	*B. subtilis* (PY79)	114	12.3±0.4	+9.9%	<0.001
glp-1(e2141)	Germ Line Proliferation	*E. coli* (OP50)	189	24.5±0.8	*B. subtilis* (PY79)	203	29.8±0.5	+22.0%	<0.0001
isp-1(qm150)	Electron transport chain	*E. coli* (OP50)	102	24.6±0.8	*B. subtilis* (PY79)	174	32.0±2.4	+30.2%	<0.0001
nuo-6(qm200)	Electron transport chain	*E. coli* (OP50)	213	31.9±0.5	*B. subtilis* (PY79)	312	38.7±1.8	+21.3%	<0.0001
eat-2(ad1116)	Dietary restriction	*E. coli* (OP50)	355	24.0±0.6	*B. subtilis* (PY79)	408	28.2±0.9	+17.3%	<0.0001
hif-1(ia4)	Hypoxia-induced factor	*E. coli* (OP50)	211	18.2±0.6	*B. subtilis* (PY79)	249	22.3±1.4	+22.1%	<0.0001
hsf-1(sy441)	Heat-shock transcription factor	*E. coli* (OP50)	107	13.1±0.3	*B. subtilis* (PY79)	112	15.1±0.4	+15.6%	<0.0005
jnk-1(gk7)	Jun N-terminal Kinase	*E. coli* (OP50)	118	15.0±0.3	*B. subtilis* (PY79)	102	21.6±0.6	+44.5%	<0.0001
dbl-1(nk3)	Transforming growth factor beta pathway	*E. coli* (OP50)	93	14.7±0.4	*B. subtilis* (PY79)	94	21.0±0.8	+42.5%	<0.0001
pmk-1(km25)	Mitogen-activated protein kinase	*E. coli* (OP50)	118	14.5±0.5	*B. subtilis* (PY79)	128	19.1±0.1	+32.0%	<0.0001
tol-1(nr2033)	Toll-like receptor	*E. coli* (OP50)	227	14.9±0.3	*B. subtilis* (PY79)	222	29.7±1.2	+98.9%	<0.0001

Table S7. Calculations for proportional life span differences of mutants with respect to controls

**C. elegans* strain	Median life span ± SD on *B. subtilis* diet	Number of life span assays (total number of worms)	**Projected median life span ± SD on *B. subtilis* diet	***p. value (T test) indicating probability that median life span is not proportionally similar to median life span of N2 controls
Wild type (N2)	23.2±1.7	13 (465)		
daf-16(mu86)	15.7±1.3	6 (229)	18.0±1.5	p<0.0001
daf-16(mgDf50)	14.9±0.3	3 (115)	19.2±0.3	p<0.005
daf-2(e1370); daf-16(mgDf50)	12.3±0.8	3 (114)	17.0±1.0	p<0.0001
glp-1(e2141)	29.8±1.1	4 (203)	21.2±0.8	p<0.05
eat-2(ad1116)	24.3±1.6	9 (408)	20.0±1.3	p<0.0005
hif-1(ia4)	22.3±2.2	6 (249)	19.8±1.9	p<0.005
hsf-1(sy441)	15.1±0.8	3 (112)	18.0±0.9	p<0.0001
pmk-1(km25)	19.1±0.2	3 (128)	20.4±0.2	p<0.05
tol-1(nr2033)	29.7±3.0	6 (222)	30.8±3.1	p<0.0001

* Strains used in Figure 6, which are not represented in this table, displayed median life spans that were proportionally similar to the median life span of N2 controls (p>0.05, T test)
** Life span data for *B. subtilis* fed mutant worms after normalizing with respect to median life span reference point obtained for N2 worms fed *E. coli* (16.0 days) and *B. subtilis* (23.2 days)
*** T test compares normalized median life spans of *B. subtilis* fed mutant worms with median life spans of *B. subtilis* fed N2 wild type worms
